# High-risk multiple myeloma predicted by circulating plasma cells and its genetic characteristics

**DOI:** 10.3389/fonc.2023.1083053

**Published:** 2023-02-09

**Authors:** Yuan Xia, Na Shen, Run Zhang, Yujie Wu, Qinglin Shi, Jianyong Li, Lijuan Chen, Min Xu, Yuanyuan Jin

**Affiliations:** ^1^ Department of Hematology, The First Affiliated Hospital of Nanjing Medical University, Jiangsu Province Hospital, Nanjing, China; ^2^ Department of Hematology, The Affiliated Taizhou People’s Hospital of Nanjing Medical University, Taizhou, China; ^3^ Department of Hematology, Zhangjiagang First Affiliated Hospital of Soochow University, Zhangjiagang, China

**Keywords:** circulating plasma cells, multiple myeloma, high-risk multiple myeloma, multi-parameter flow cytometry, gene mutation, next-generation sequencing

## Abstract

**Introduction:**

Circulating plasma cells (CPC) have been reported to be one of the indicators of high-risk multiple myeloma (MM), yet the prognostic significance of CPC in Chinese population and the genetic mechanisms underlying CPC formation have not been fully elucidated.

**Methods:**

Patients with newly diagnosed MM were included in this study. We used multi-parameter flow cytometry (MFC) for CPC quantification and next-generation sequencing (NGS) technology for mutational landscape mapping to identify the correlation of CPC level with clinical characteristics and the mutations.

**Results:**

A total of 301 patients were enrolled in this investigation. We demonstrated that CPC quantification could effectively mirror the tumor load, and CPC ≥ 0.105% at diagnosis or detectable CPC after therapy indicates poor treatment response and adverse outcome, and the introduction of CPC into the R-ISS enables a more accurate risk stratification. Interestingly, we noticed an elevated percentage of light-chain MM in patients with higher CPC. Mutational landscape revealed that patients harboring mutations in TP53, BRAF, DNMT3A, TENT5C, and IL-6/JAK/STAT3 pathway-related genes tended to have higher CPC levels. Gene enrichment analysis demonstrated that pathways involving chromosome regulation and adhesion may be potential mechanisms accounting for CPC formation.

**Discussion:**

Accordingly, quantification of CPC may provide a less-invasive and reliable approach for identifying high-risk MM in Chinese population.

## 1 Introduction

Multiple myeloma (MM), one of the most common hematological malignancies, is caused and characterized by clonal proliferation of malignant plasma cells (1). Clonal plasma cells are predominantly distributed in the bone marrow (BM), mainly due to their adhesion to the BM microenvironment (2, 3). Along with the technical improvement, it has become clear that clonal plasma cells not merely reside in the BM but can also passage into the circulation and subsequently home to intramedullary or distant tissues, a process that allows clonal plasma cells to circulate and reside in the peripheral blood, and these cells are named circulating plasma cells (CPC) (3).

The rise of multi-parameter flow cytometry (MFC) has brought reliable methods for diagnosis and minimal residual disease (MRD) monitoring in MM, and, at the same time, detection of minimal CPC in the peripheral blood (4, 5). Moreover, detection of CPC by MFC could eliminate the need for repeated invasive BM biopsies and possess higher sensitivity than conventional slide-based methods (6). Elevated CPC has been shown in previous studies to suggest a poor prognosis (7–9), while most studies on CPC were conducted in Caucasian populations. Given the racial and therapeutic heterogeneity, the prognostic implication of CPC quantification, especially its dynamics, has not been fully elucidated in Chinese population.

The mutational landscape is currently one of the most intensively studied fields in MM. It was reported that an average of 1.6 mutations were detected per Mb in MM (10). High-throughput DNA sequencing, also known as next-generation sequencing (NGS), has been widely used in MM for the past few years, and mutations like KRAS, NRAS, TP53, FAM46C and DIS3 were observed recurrently. Yet, mutational landscape was heterogeneous in different studies (11–13), and the relationship between mutations and CPC levels in Chinese population has not been well explored (14–17). Previous studies have shown high concordance in gene expression between bone marrow plasma cells (BMPC) and CPC by NGS technology (14, 16), suggesting that CPC are mainly derived from tumor cells in the BM. In this work, NGS was applied to molecularly characterize the BM myeloma cells to determine the association of CPC levels with the mutational landscape.

Here, we used MFC for CPC quantification and NGS technology for mutational landscape mapping to verify the implication of CPC in risk stratification, and to further elucidate the relationship between genetic alteration and CPC quantification. To the best of our knowledge, this is one of the most extensive studies in Chinese population to date focusing on the clinical features and the underlying genetic characteristics regarding CPC.

## 2 Materials and methods

### 2.1 Study population

A total of 301 patients with newly diagnosed multiple myeloma (NDMM) admitted to the First Affiliated Hospital of Nanjing Medical University between October 2015 and May 2021 were enrolled. Diagnosis and response assessment of MM were performed according to the revised International Myeloma Working Group (IMWG) criteria (18, 19). Patients with plasma cell leukemia were excluded. All patients were followed up until October 2021, with a median follow-up time of 29 months (range, 1-70 months). This retrospective study was conducted in accordance with the Declaration of Helsinki, and was approved by the institutional review boards of the First Affiliated Hospital of Nanjing Medical University Ethics Committee (No. 2020-SR-589). Informed consents were obtained from all patients before enrollment.

### 2.2 Multi-parameter flow cytometry

Samples of blood or BM were stained with antibodies to CD19, CD27, CD38, CD45, CD56, CD138, cLambda and cKappa, and cells were subsequently detected by 8-color flow cytometry to quantify CPC and BMPC. Details of the flow cytometry technique for CPC and BMPC detection have been described in our previous studies (20, 21).

### 2.3 Next-generation sequencing and analysis of mutations

NGS technology and analysis of mutations have been described in detail in our previous study (22). In brief, BM aspirates were obtained at diagnosis and were sorted by anti-CD138 magnetic microbeads for the purification of tumor cells. Genomic DNA (gDNA) was extracted from tumor cells using DNA extraction kits (QIAGEN, Germany) according to the manufacturer’s instructions, and the gDNA was fragmented by Enzyme Plus Library Prep Kit (iGenetech, China). Libraries were prepared using probes and TargetSeq One Kit (iGenetech, China) and were subjected to NovaSeq 6000 platform (Illumina, USA) for sequencing, targeting 387 genes involving pan-cancer driver genes and MM-related genes (genes panel is listed in **Supplementary Table 1**), with an average sequencing depth of 1,000×. Reads were aligned and data were subsequently converted and filtered. Mutations were annotated, analyzed and visualized by R package “maftools” (23).

### 2.4 Gene enrichment analysis

Gene Ontology (GO) enrichment comprising “Biological Process (BP)”, “Cellular Component (CC)”, and “Molecular Function (MF)”, as well as Kyoto Encyclopedia of Genes and Genomes (KEGG) enrichment was applied to uncover the biological functions and pathways of gene clusters. Gene enrichment and visualization were conducted using R package “clusterProfiler” (24). GO terms and KEGG pathways with *P*<0.05 based on the cumulative hypergeometric distribution test were significantly enriched.

### 2.5 Statistical analysis

Mann-Whitney U or Kruskal-Wallis tests were used for analyzing continuous variables, and χ2 or Fisher’s exact tests were used for comparing categorical data. Spearman’s rank correlation coefficient (r_s_) was used to estimate the correlation between variables. The optimal cut-off value was determined by the Youden index based on the receiver operating characteristic (ROC) curve. Progression-free survival (PFS) and overall survival (OS) were plotted as Kaplan-Meier curves and were compared by log-rank test using R package “survival” and “survminer”. Data were analyzed by SPSS (v23.0, IBM Corp.) and R software (v4.1.1, R Foundation for Statistical Computing). All tests were two-sided, and a *P <*0.05 was considered statistically significant.

## 3 Results

### 3.1 Relationship between pre-therapeutic CPC level and clinical characteristics

A total of 301 patients with NDMM were enrolled, including 178 males and 123 females, with a median age of 56 years (range, 30-84 years). To determine the optimal cut-off value for CPC, the ROC curves were analyzed and the cut-off value of 0.105% with the highest Youden index were selected. To investigate the relationship between pre-therapeutic CPC and clinical features, patients were divided into CPC-low and CPC-high groups according to their CPC levels by the cut-off value of 0.105%. As shown in **Table 1**, CPC-high patients had significantly lower hemoglobin (*P*=0.0025) and higher lactate dehydrogenase (LDH) (*P*=0.0028) and β2-microglobulin (*P*<0.0005). Significantly more patients in the CPC-high group presented stage III disease, according to both International Staging System (ISS) and revised ISS (R-ISS) (*P*=0.0007 and =0.0003, respectively). According to the latest R2-ISS (25), in which patients were stratified into for risk groups, more patients in CPC-high group were stratified as R2-ISS IV than those in CPC-low group (*P*=0.0098). It is interesting to note a different subtype pattern between groups, with a higher proportion of light-chain subtype (*P*=0.0026) and a lower proportion of IgA subtype (*P*=0.0153) in the CPC-high group.

**Table 1:** Clinical characteristics of patients according to quantification of CPC.

Clinical characteristics	CPC-low (n=210)	CPC-high (n=91)	*P*
Age, years, median (IQR)	63 (54.3-68)	63 (56-67)	0.672
Sex, n (%)			0.762
Male	123 (58.6)	55 (60.4)	
Female	87 (41.4)	36 (39.6)	
Subtype, n (%)			**0.0084**
IgG	101 (48.1)	40 (44.0)	
IgA	57 (27.1)	13 (14.3)	
Light chain	40 (19.1)	32 (35.2)	
Other	12 (5.7)	6 (6.59)	
D-S, n (%)			0.2392
I	12 (5.7)	2 (2.2)	
II	34 (16.2)	11 (12.1)	
III	164 (78.1)	78 (85.7)	
ISS, n (%)			**0.0011**
I	38 (18.1)	8 (8.8)	
II	86 (41.0)	25 (27.5)	
III	86 (41.0)	58 (63.7)	
R-ISS, n (%)^a^			**0.0023**
I	24 (12.7%)	6 (6.74%)	
II	135 (71.43%)	53 (59.55%)	
III	30 (15.87%)	30 (33.71%)	
R2-ISS, n (%)^a^			**0.0066**
I	14 (7.78%)	1 (1.14%)	
II	38 (21.11%)	12 (13.64%)	
III	110 (61.11%)	56 (63.64%)	
IV	18 (10%)	19 (21.59%)	
Hemoglobin, g/L, median (IQR)	93 (73-111.8)	81 (68-98)	**0.0025**
Albumin, g/L, median (IQR)	33.2 (28.4-38.4)	32.5 (26.3-37.7)	0.2782
LDH, U/L, median (IQR)	166 (139-211)	192 (151-252)	**0.0028**
Creatinine, μmol/L, median (IQR)	78.2 (58.9-113.8)	81.8 (65.9-188.8)	0.0759
Corrected calcium, mmol/L, median (IQR)	2.4 (2.3-2.5)	2.4 (2.3-2.6)	0.2501
β2-microglobulin, mg/L, median (IQR)	4.2 (3-7.9)	6.8 (3.8-13.2)	**<0.0005**
BMPC (morphology), %, median (IQR)	17 (10.4-30.7)	26.8 (13.6-51.8)	**<0.0005**
BMPC (MFC), %, median (IQR)	4.2 (1.2-9.4)	14.6 (6.5-25.1)	**<0.0005**
EMD, n (%)	52 (24.8)	17 (18.7)	0.2491
Chomosomal abnormality, n (%)^a^			
gain(1q)	89 (49.4)	49 (55.7)	0.3373
del(17p)	15 (8.3)	18 (20.5)	**0.0046**
t(4;14)	29 (16.1)	18 (20.5)	0.3799
t(11;14)	13 (7.2)	7 (8.0)	0.8304
t(14;16)	1 (0.6)	2 (2.3)	0.2518
Induction therapy			0.0610
PI-based	78 (37.14%)	22 (24.18%)	
IMiD-based	99 (47.14%)	45 (49.45%)	
PI+IMiD	29 (13.81%)	20 (21.98%)	
Others	4 (1.9%)	4 (4.4%)	
Response^a^			
PR or better	179 (91.8)	59 (74.7)	**0.0001**
VGPR or better	162 (83.1)	44 (55.7)	**<0.0005**
CR or better	112 (57.4)	30 (38.0)	**0.0035**
MRD negative after treatment^a^	124 (44.57%)	31 (33.33%)	**0.0006**

a Assessed on available data. Bold values indicate statistical significance (P<0.05).

CPC, circulating plasma cells; IQR, interquartile range; D-S, Durie-Salmon staging system; ISS, International Staging System; R-ISS, revised International Staging System; BMPC, bone marrow plasma cells; MFC, multi-parameter flow cytometry; LDH, lactate dehydrogenase; EMD, extramedullary disease; PI, proteasome inhibitors; IMiD, immunomodulatory drugs; PR, partial response; VGPR, very good partial response; CR, complete response.

**Figure 1:**
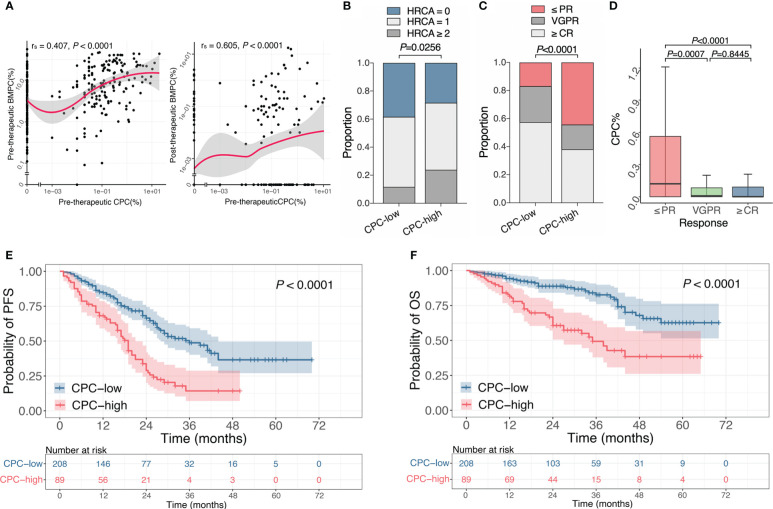
Association of CPC with clinical features, efficacy and prognosis at initial diagnosis. **(A)** The Spearman’s correlation between pre-therapeutic CPC and BMPC before and after treatment. **(B)** Proportion of different HRCA counts in the CPC-low and -high group. **(C)** Response rates of patients after induction therapy in the CPC-low and -high group. **(D)** CPC levels in patients with different states of remission. **(E, F)** Kaplan-Meier curves for PFS **(E)** and OS **(F)** of patients with different CPC levels. CPC, circulating plasma cells; BMPC, bone marrow plasma cells; HRCA, high-risk chromosomal abnormality; PR, partial response; VGPR, very good partial response; CR, complete response; PFS, progression free survival; OS, overall survival.

Both morphology and MFC methods showed higher levels of BMPC in the CPC-high group (**Table 1**). We discovered a significant correlation between BMPC and CPC at diagnosis (r_s_=0.407, *P*<0.0001) (**Figure 1A**, left). Interestingly, we also noticed a significant correlation between pre-therapeutic CPC and BMPC after induction therapy (r_s_=0.605, *P*<0.0001) (**Figure 1A**, right).

Cytoplasmic light chain immunofluorescence with fluorescence *in situ* hybridization (cIg-FISH) was performed in 268 patients. Patients in the CPC-high group showed a higher incidence of del(17p) (*P*=0.0046), whereas no differences were observed for other chromosomal abnormalities (**Table 1**). According to the latest Mayo Stratification of Myeloma and Risk-Adapted Therapy (mSMART) (26) and Mayo Additive Staging System (MASS) (27), t(4;14), t(14:16), t(14;20), del(17p) and gain(1q) are defined as high-risk chromosomal abnormality (HRCA). We discovered a statistical difference in HRCA counts between the two groups, with 38.3%, 50% and 11.7% of patients in the CPC-low group that harbored 0, 1 or ≥2 HRCA(s), while it was respectively 28.4%, 47.7% and 23.9% in the CPC-high group (*P*=0.0256) (**Figure 1B**).

### 3.2 Predictive value of pre-therapeutic CPC level for efficacy and prognosis

Treatment responses were assessed after induction therapy, and clinical data of 274 patients were available for response evaluation. Response rates of ≥ partial response (PR), ≥ very good partial response (VGPR) or ≥ complete response (CR) in CPC-high group were significantly lower than those in the CPC-low group (*P*<0.05) (**Table 1** and **Figure 1C**). Similarly, patients with the responses of ≤PR had significantly higher CPC levels than those with VGPR or ≥CR (*P*=0.0007 and *P*<0.0001, respectively), while no difference was observed between patients with responses of VGPR and ≥CR (**Figure 1D**). It is found that patients in the CPC-low group have a higher rate of MRD negativity. The median PFS of the CPC-low and CPC-high groups were 36 months and 19 months, and the median OS was not reached and 35 months, respectively (*P*<0.0001 for both PFS and OS). Higher levels of CPC retained adverse effects on PFS and OS in NDMM (**Figures 1E, F**).

### 3.3 Predictive value of post-therapeutic CPC level for efficacy and prognosis

Of the 144 patients with detectable CPC at diagnosis and with clinical data available for efficacy assessment, CPC turned negative in 124 patients (86.1%) during induction therapy and remained positive in the rest 20 patients (13.9%), for whom the median CPC was 0.195% (range, 0.002%-3.25%).

**Figure 2:**
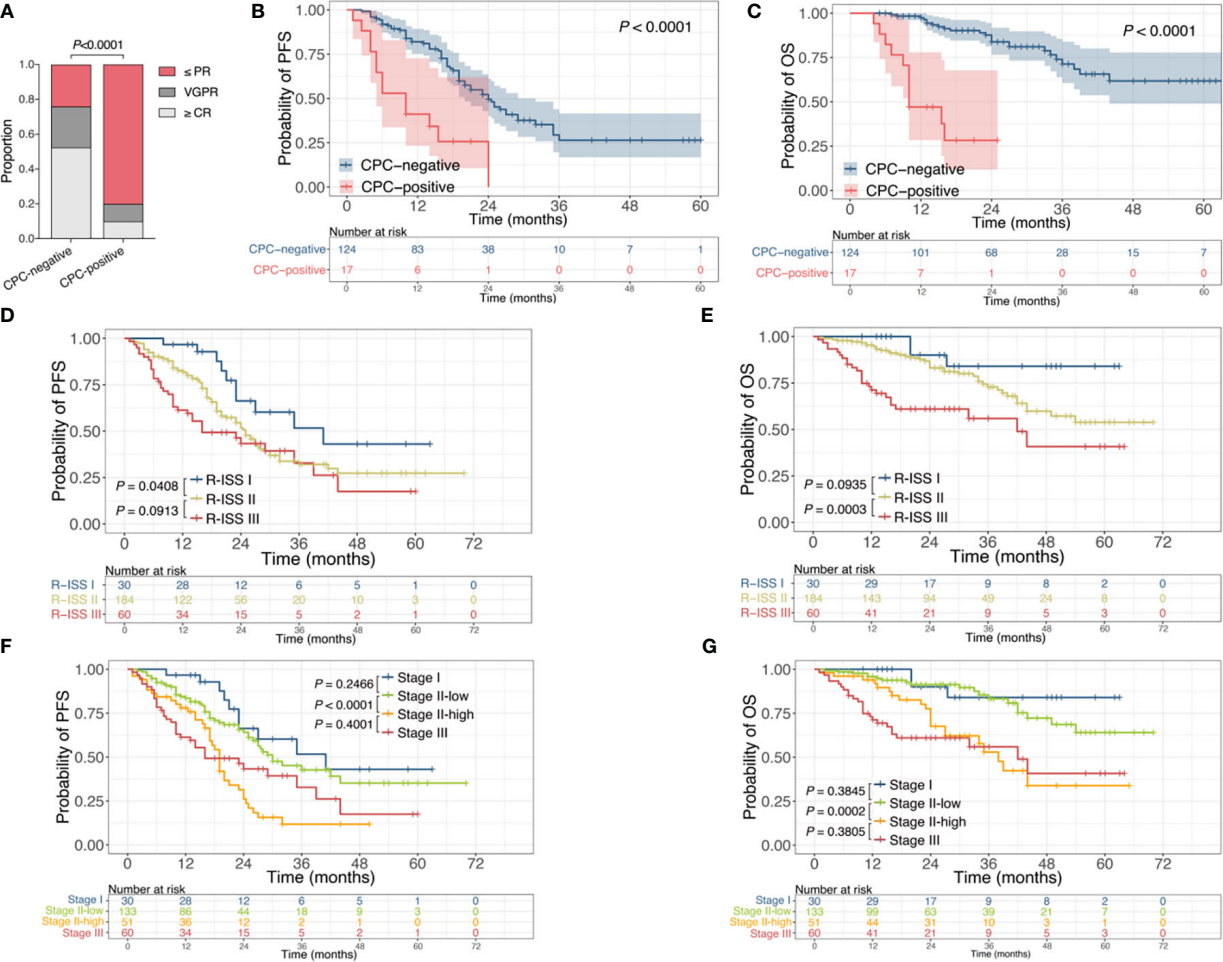
Association of post-therapeutic CPC with efficacy and prognosis. **(A)** Response rates of patients with or without remaining CPC after induction therapy. **(B, C)** Kaplan-Meier curves for PFS **(B)** and OS **(C)** of patients with or without remaining CPC after induction therapy. **(D, E)** Kaplan-Meier curves for PFS **(D)** and OS **(E)** stratified by R-ISS. **(F, G)** Kaplan-Meier curves for PFS **(F)** and OS **(G)** stratified by R-ISS combined with CPC levels. CPC, circulating plasma cells; PR, partial response; VGPR, very good partial response; CR, complete response; R-ISS, revised International Staging System; PFS, progression free survival; OS, overall survival.

To investigate the implication of post-therapeutic CPC, patients were grouped into CPC-negative and CPC-positive subgroups, with the former referring to patients with undetectable CPC after induction therapy and the latter denoting the ones with remaining CPC when induction therapy finished. The response rates of ≤PR, =VGPR, and ≥CR were 24.2%, 23.4% and 52.4% in the CPC-negative group, and were 80%, 10% and 10% in the CPC-positive group (**Figure 2A**), illustrating a worse efficacy conveyed by detectable post-therapeutic CPC (*P*<0.0001). Moreover, detectable post-therapeutic anticipated poor outcome in MM, with the median PFS of 24 months vs 8.75 months (*P*<0.0001) and the median OS of not reached vs 11.5 months (*P*<0.0001) for the CPC-negative and CPC-positive subgroups, respectively (**Figures 2B, C**).

### 3.4 Implication of CPC in improving the accuracy of prognostic discrimination of R-ISS

The R-ISS is one of the most recognized and widely used staging systems for risk stratification in MM. For the entire cohort, the R-ISS could predict the outcomes in general (P=0.0037 for PFS, P<0.0001 for OS), yet failed to distinguish the difference of PFS between R-ISS II and III, nor the difference of OS between R-ISS I and II (**Figures 2D, E**). Since up to 67.6% of patients were R-ISS II in this study, to evaluate the significance of CPC as a further biomarker for prognosis prediction, we introduced the CPC level into the R-ISS. Patients with R-ISS II disease were further grouped by CPC quantification, with those who had CPC<0.105% at diagnosis defined as Stage II-low and the rest as Stage II-high. Patients with R-ISS I and III disease were defined as Stage I and Stage III, respectively. The median PFS for Stage I, Stage II-low, Stage II-high, and Stage III were 41 months, 30 months, 19 months, and 16 months, and the median OS were not reached, not reached, 38 months, and 42 months, respectively (**Figures 2F, G**). Accordingly, the introduction of CPC levels into the R-ISS demonstrate more robust discrimination of prognosis for NDMM, especially for the larger number of R-ISS II patients.

### 3.5 Association between mutations and CPC levels

**Figure 3:**
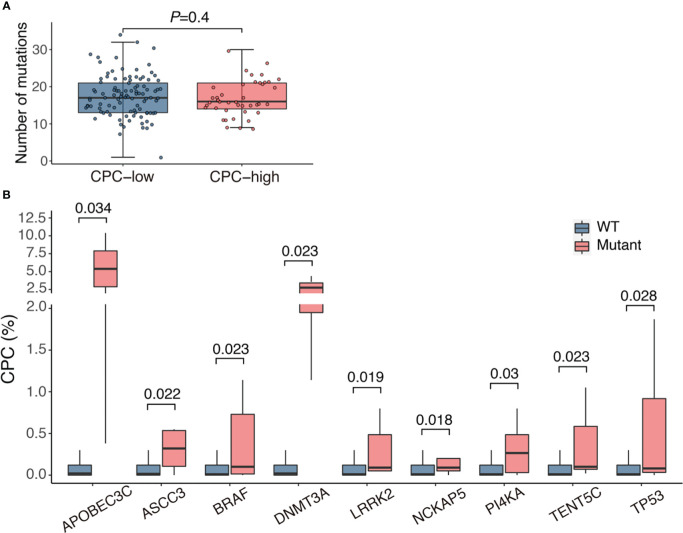
Association of CPC level with mutational burden and gene mutations. **(A)** Mutational load in patients with different CPC levels. **(B)** Comparison of CPC levels between patients with mutations and their wild-type counterparts. CPC, circulating plasma cells; WT, wild type.

NGS was performed in 143 patients, and mutations were detected in all patients, involving 337 genes (The mutations of each patients were listed in **Supplementary Table 2**). The median number of mutations was 17 (range, 1-34), and the median number of genes involved was 15 (range, 1-28), with the most frequently mutated genes being KRAS (29.4%), NRAS (23.1%), IGLL5 (19.6%), SYNE1 (18.9%) and AHNAK2 (17.5%). There was no statistical difference in the number of mutations between CPC-low and CPC-high patients, with a median of 17 (range, 1-34) in the former group and 16 (range, 9-30) in the latter one (**Figure 3A**). We compared the CPC levels of patients with specific mutations to their wild-type counterparts and found that patients who bore mutations involving APOBEC3C, ASCC3, BRAF, DNMT3A, LRRK2, NCKAP5, PI4KA, TENT5C and TP53 tended to have significantly higher CPC levels (**Figure 3B**).

### 3.6 Biological functions and pathways associated with CPC levels

**Figure 4:**
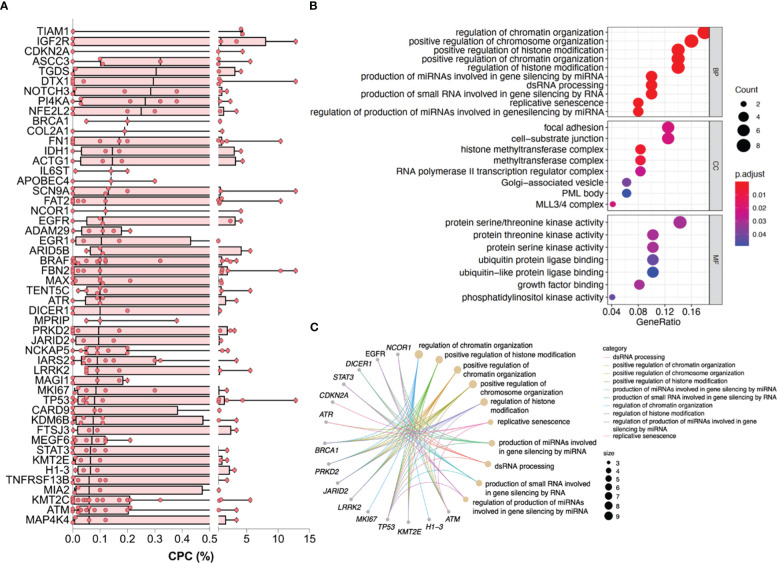
Genes and enriched pathways associated with higher CPC levels. **(A)** The top 50 genes with the highest CPC levels. **(B)** Dotplot reflecting top terms in GO enrichment. **(C)** Cnetplot of pathway-gene network for the top 10 terms of GO enrichment and relevant genes. CPC, circulating plasma cells; GO, Gene Ontology.

For the exploration of underlying biological functions or pathways involved in the CPC elevation, we eliminated mutations that were only presented in ≤2 patients and ranked the remaining mutations by the median CPC level. The mutations with the highest CPC level involved several genes that have received attention in MM, such as TP53, ATM, BRAF, IL6ST, EGFR, STAT3, PRKD2, and MKI67. The top 50 genes with the highest CPC levels are shown in **Figure 4A**.

Enrichment was subsequently performed. GO terms regarding the modification and regulation of chromosome, which contains DNA, RNA, histones, etc., were significantly enriched in BP and CC categories. Pathways associated with adhesion like focal adhesion and cell-substrate junction were statistically enriched in the CC category. The enriched MF terms were mainly involved in the catalytic activity of energy metabolism (**Figure 4B**). The top 10 terms of the GO enrichment and their relevant genes, like TP53, ATM, EGFR, and BRCA1, were presented as a pathway-gene network in **Figure 4C**. The enriched KEGG pathways were mainly of tumor signaling pathways (**Figure S1**).

## 4 Discussion

The past decade has witnessed an ever-increasing focus on CPC, and the extensive application of MFC in CPC detection allows easy and non-invasive examination of tumor cells (6, 7, 28). To investigate the implication of CPC on tumor burden and its predictive value for efficacy and prognosis, we conducted this clinical study involving 301 patients, one of the most extensive clinical studies to date. As a result, we found that higher CPC was associated with lower hemoglobin and higher β2-microglobulin and LDH. The β2-microglobulin, LDH and hemoglobin are well-documented biomarkers of tumor load in MM, thus suggesting the presence of CPC to be a result of high disease burden. BMPC is one of the most established parameters for the diagnosis and monitoring of MM, which involves invasive and painful procedures. Korthals et al. (29) reported that the MRD level of peripheral blood was 40-fold lower than their BM counterparts when detected by IgH-PCR. A non-linear correlation was found between absolute CPC count and BMPC percentage in another study using MFC (30). In the present work, we validated that higher CPC was associated with elevated BMPC by both morphological and MFC method. We also observed a significant positive correlation between BMPC and CPC (both by MFC). Therefore, detection of CPC could be used to monitor tumor load without the need for repeated BM biopsy.

It has been addressed that CPC quantification were correlated with the prognosis of NDMM (7) and relapsed/refractory MM (8, 9). In this work, we found that high levels of pre-therapeutic CPC suggest poor response and adverse outcome in NDMM. It is noteworthy that the presence of post-therapeutic CPC indicated a low response rate, with up to 80% of patients having a response of PR or worse. In contrast, for patients with undetectable CPC after treatment, 75.8% achieved remission of ≥VGPR. Similarly, the remaining CPC portended a dismal prognosis with a median OS of only 11.5 months. Hence, the dynamics of CPC may be a promising biomarker to identify high-risk MM.

MM is a heterogeneous disease with diversified outcomes (31). The R-ISS is the most recognized system for prognostic categorizing (32), but up to half of the patients are classified as R-ISS II, and the prognosis of these patients varies widely. A study from Mayo Clinic showed a similar prognosis between R-ISS I/II patients with ≥5 CPCs/μL and R-ISS III patients (33). When the CPC quantification was introduced into the existing R-ISS, we were excited to observe a more robust risk stratification for the large number of patients with R-ISS II disease, signifying the potential role of CPC in more accurate prognostic anticipation.

Interestingly, we discovered a higher proportion of light-chain subtype in CPC-high patients than the CPC-low ones, which, to the best of our knowledge, is the first report to date describing the subtype pattern regarding different CPC levels. This phenomenon was highly similar to that observed in plasma cell leukemia (PCL) (34, 35), which represents one of the most aggressive forms of MM and typically denoted a dismal outcome even in the era of novel agents (34). It is known that a complete immunoglobulin is composed of heavy and light chains. The immunoglobulin heavy chain (IgH) is encoded by the IGH gene located on chromosome 14q32 (36), and the translocation of IgH, considered the primary genetic event of MM, occurs in 45% of MM patients (26) and more than 80% of PCL patients (35). In this work, we observed a higher rate of IgH translocation in CPC-high MM than the CPC-low MM. Since IgH translocation could yield the deletion or silence of the IGHV gene necessary for IgH assembly (37), we speculated that the higher incidence of IgH translocation in CPC-high MM and PCL potentially lead to the impaired heavy chain synthesis, and hence, the higher rate of light-chain subtype. Additionally, we noted that del(17p) occurred more frequently in CPC-high MM than that in CPC-low MM (20.5% *vs* 8.3%); and at the same time, Gundesen et al. (34) demonstrated a higher incidence of del (17p) in PCL that in MM (40% *vs* 11%). Tumor cells in PCL are generally considered to express higher levels of CD19, CD20 and CD45 than those in MM (38, 39); similarly, upward expression of these biomarkers were observed in CPC than their BM counterparts (30). The elevated expression of these antigens may represent, according to the phenotypic transformation of B-cell development (40), a poorly-differentiated cell population that is believed to suggest inferior survival in MM (41). A Chinese study by An et al. (42) illustrated that MM patients with a small amount of CPC had similar prognosis to PCL. Accordingly, the presence of CPC≥0.105%, a threshold represents even a minimal amount of CPC, represents a relatively aggressive MM, and we speculate that the similar phenotypes and cytogenetic characteristics exhibited by CPC-high MM and PCL may partly explain their congruent subtype pattern and adverse outcome.

To further elucidate the association of CPC level with genetic features, NGS was employed to characterize the tumor cells. To our knowledge, this is the first time that NGS has been used to molecularly characterize the tumor cells in a study of CPC in Chinese population. As a result, while more aggressive myeloma is thought to have a high mutational burden (43), we found no significant differences in mutational burden between the CPC-low and CPC-high groups. When CPC levels were compared between patients who carried WT and mutant genes, patients with mutations involving TP53, BRAF, DNMT3A, APOBEC3C, ASCC3 and TENT5C, *etc.*, tended to have significantly higher CPC levels. We have noticed that TP53 mutation, one of the most studied mutations in MM, was related to a higher level of CPC, in line with the superior frequency of del(17p) in CPC-high patients by FISH method. TP53 mutation was reported to be associated with the migration of MM cells from the BM into peripheral blood, thereby facilitating the development of PCL (44, 45). Lee et al. (45) reported that mutation of TP53 was more frequent in PCL than in MM. These findings emphasize the role of TP53 in prompting the MM cells to migrate into the blood.

It is interesting that mutations of IL6ST, EGFR and STAT3 suggested enhanced CPC levels. A latest investigation for functional high-risk (FHR) MM, which refer to patients with suboptimal response to induction therapy or early relapse, demonstrated increased mutations in the IL-6/JAK/STAT3 pathway in FHR patients (46). Therefore, elevated CPC may portend the possibility of FHR MM, providing a simple alternative for the identification of high-risk MM.

To unveil the underlying pathways involved in the egress of MM cells towards the circulation, we performed enrichment for the mutant genes with the highest CPC levels. As a result, pathways involving the regulation of chromosome as well as adhesion and junction were significantly enriched. It has been reported that the CPC represent a subset of myeloma cells with downregulated integrins and adhesion molecules and thus tended to become a BM microenvironment independent subpopulation (14, 47); our results validate the role of adhesion and junction in CPC formation at the genetic level. The regulation of chromosome involves plenty of physiological processes, of which chromosomal instability is most extensively studied in MM. Chromosomal instability can result in copy number and structural changes of chromosomes, and act as a critical element in the development and progression of MM (43, 48), whereas the relationship between CPC and chromosomal instability as well as other procedures involved in chromosome regulation remains poorly understood. Our findings proposed a suggested mechanism of CPC formation, and additional efforts are still warranted.

There are still some limitations in this study. The tumor cells subjected to NGS were sorted by the positive expression of CD138. Although CD138 is well recognized to be expressed on MM cells (49), it has been recently reported that few MM clones lacks the expression of CD138 and these CD138-negative cells may represent a group of cancer stem cells with a higher migration capacity (50, 51). Therefore, the application of NGS in combination with highly purified fluorescence-activated cell sorting (FACS) based on the specific aberrant phenotypes of patients or single-cell RNA sequencing may provide a more accurate method to characterize MM cells. In addition, limited to current technologies, the sensitivity of MFC is insufficient to detect CPC in a small half of the patients; hence, the detection of BM aspirates is still the gold standard for MRD monitoring, especially for patients with preferable responses. NGS technology has been proved to be extremely sensitive in CPC detecting (16), but the prohibitive cost limits its application in disease assessment. Therefore, more sensitive flow cytometry, as well as less expensive sequencing techniques, are still warranted.

## 5 Conclusion

To conclude, we adopted the emerging technologies, comprising MFC and NGS, to analyze the correlation of CPC with clinical characteristics and gene mutations. We demonstrated that CPC level can effectively reflect the tumor load, and high level of CPC at diagnosis or after induction therapy can predict high-risk MM that indicate poor treatment response and adverse outcome. We present the phenomenon and mechanistic speculation of an elevated proportion of light-chain subtype in patients with higher CPC. Analysis of the mutational landscape revealed that TP53 mutation and pathways involving chromosome regulation and adhesion may be the underlying mechanism of CPC formation. Overall, while bone marrow examination remains the gold standard in MRD detection for most MM patients, the detection of CPC by MFC may provide a less-invasive, convenient and, more importantly, reliable approach for risk stratification and disease monitoring. Large-scale clinical research and further in-depth mechanism explorations are required for a better understanding of CPC, thus, shed light on promising therapies to overcome the adverse outcome associated with CPC.

## Data availability statement

The original contributions presented in the study are included in the article/**Supplementary Material**. Further inquiries can be directed to the corresponding author.

## Ethics statement

Ethical review and approval was obtained from the institutional review boards of the First Affiliated Hospital of Nanjing Medical University Ethics Committee (No. 2020-SR-589). Informed consents were obtained from all patients before enrolment.

## Author contributions

YX designed the study. YX and NS collected the data. YX and MX analyzed and interpreted the data. YX drafted the manuscript, YJ and LC revised the manuscript. All authors contributed to the article and approved the submitted version.
