# Supplementary material for: High-risk multiple myeloma predicted by circulating plasma cells and its genetic characteristics
Source: Front Oncol. 2023 Feb 9;13:1083053. doi: 10.3389/fonc.2023.1083053 (PMC9947848; doi:10.3389/fonc.2023.1083053)

Supplementary Material

# Supplementary Table 1. Gene panel for next-generation sequencing.

# Supplementary Table 2. The quantification of CPC and the mutations of each patient.

# Supplementary Figure 1. Dotplot of KEGG signal pathway enrichmen.

#
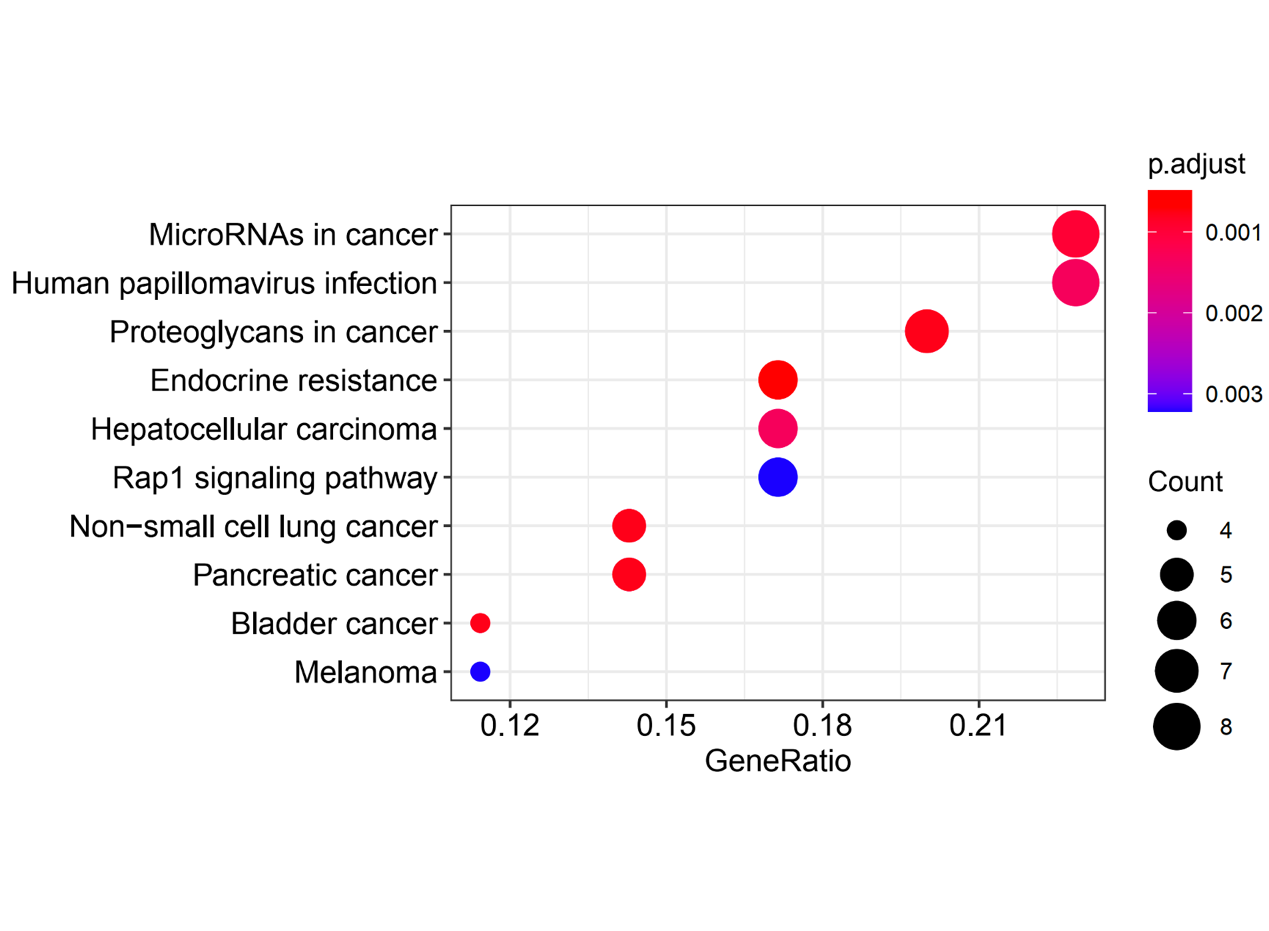

Supplement: Supplementary file 1 [file DataSheet_1.doc]
